# MP29-02* reduces eosinophil survival induced by epithelial cell secretions from nasal mucosa

**DOI:** 10.1186/2045-7022-5-S4-P9

**Published:** 2015-06-26

**Authors:** Jordi Roca-Ferrer, Laura Pujols, Maria Pérez-Gonzalez, Isram Alobid, Antonio Valero, Cesar Picado, Ruth Murray, Joaquim Mullol

**Affiliations:** 1 IDIBAPS & CIBERES, Barcelona, Spain; 2 IDIBAPS, CIBERES & Universitat de Barcelona, Barcelona, Spain; 3 Medscript, Dundalk, Ireland

## Background

Recently, MP29-02* (a novel intranasal formulation of azelastine hydrochloride [AZ] and fluticasone propionate [FP]) has demonstrated significant clinical effects in AR compared to these drugs in monotherapy. The aim of this study was to investigate the anti-inflammatory effect of MP29-02* compared to AZ and FP alone in an in vitro validated model of eosinophilic inflammation.

## Methods

Peripheral blood eosinophils were incubated for 4 days with decreasing dilutions of MP29-02* (from 1:10^2^ to 1:10^5^ times), equivalent dilutions of FP (7.3x10^-6^M to 10^-9^M) or AZ (2.4x10^-5^M to 10^-8^M) prior to the addition of Epithelial Cell culture Media (ECM) from nasal mucosa (NM). Eosinophil survival was assessed by Trypan blue dye exclusion. Results are expressed as percentage (mean ± SEM) of eosinophil survival compared to control (100%).

## Results

ECM from NM at 10% induced eosinophil survival from day 1 to 4. This effect was inhibited in a dose-response manner by MP29-02* and FP alone (from day 2 to 4) and AZ alone (only at day 4). At day 3, MP29-02* significantly inhibited eosinophil survival induced by ECM from dilution 1:10^2^ (13.8±1.5%, N=6) to dilution 1:10^5^ (58.8±10.8%, N=6), compared to ECM (100%). This inhibitory effect on eosinophil survival induced by MP29-02* at 1:10^2^ dilution (13.8±1.5%) was significantly (p<0.05) stronger than that induced by FP alone (36.7±6.3) or AZ alone (70.3±10.4%) at similar dilutions.

## Conclusions

These results suggest that MP29-02* may reduce upper airway eosinophilic infiltration more potently than corticosteroids or antihistamines administered alone. This anti-inflammatory effect may account, at least in part, for the stronger clinical effect of MP29-02* on moderate to severe allergic rhinitis when compared to these drugs in monotherapy.

This study has been sponsored by a research grant from MEDA Pharma.

* Dymista

